# Investigation of Intestinal Microbiota and Short-Chain Fatty Acids in Colorectal Cancer and Detection of Biomarkers

**DOI:** 10.3390/pathogens14090953

**Published:** 2025-09-22

**Authors:** Esra Saylam, Özben Özden, Fatma Hümeyra Yerlikaya, Abdullah Sivrikaya, Serdar Yormaz, Uğur Arslan, Mustafa Topkafa, Salih Maçin

**Affiliations:** 1 Department of Medical Microbiology, Faculty of Medicine, Selçuk University, Konya 42130, Turkey; emeterelliyoz@gmail.com (E.S.); drarslanugur@gmail.com (U.A.); 2 Department of Medical Biotechnology, Institute of Health Sciences, Acıbadem University, Istanbul 34752, Turkey; ozbenozdennn@gmail.com; 3 Department of Medical Biochemistry, Faculty of Medicine, Selçuk University, Konya 42130, Turkey; fhumeyray@hotmail.com (F.H.Y.); biyokaya@selcuk.edu.tr (A.S.); 4 Department of General Surgery, Faculty of Medicine, Selçuk University, Konya 42130, Turkey; serdaryormaz@selcuk.edu.tr; 5 Department of Chemistry and Chemical Processing Technology, Konya Technical University, Konya 42250, Turkey; mtopkafa@ktun.edu.tr

**Keywords:** colorectal cancer, biomarker, metagenomics, microbiota, next generation sequencing, short-chain fatty acids, Zonulin

## Abstract

Colorectal cancer (CRC) is one of the most common cancers worldwide and a significant global health issue. The human gut microbiota, a complex ecosystem hosting numerous microorganisms such as bacteria, viruses, fungi, and protozoa, plays a crucial role. Increasing evidence indicates that gut microbiota is involved in CRC pathogenesis. In this study, the gut microbiota profiles, short-chain fatty acids, zonulin, and lipopolysaccharide-binding protein levels of newly diagnosed CRC patients were analyzed along with healthy controls to elucidate the relationship between CRC and the gut microbiota. The study included 16 newly diagnosed CRC patients and 16 healthy individuals. For microbiota analysis, DNA isolation from stool samples was performed using the Quick-DNA™ Fecal/Soil Microbe Miniprep Kit followed by sequencing using the MinION device. Data processing was conducted using Guppy software (version 6.5.7) and the Python (3.12) programming language. ELISA kits from Elabscience were utilized for analyzing LBP and zonulin serum levels. Fecal short-chain fatty acids were analyzed using GC-MS/MS equipped with a flame ionization detector and DB-FFAP column. Microbial alpha diversity, assessed using Shannon and Simpson indices, was found to be lower in CRC patients compared to healthy controls (*p* = 0.045, 0.017). Significant differences in microbial beta diversity were observed between the two groups (*p* = 0.004). At the phylum level, *Bacteroidota* was found to be decreased in CRC patients (*p* = 0.027). Potential biomarker candidates identified included Enterococcus faecium, *Ruminococcus bicirculans*, *Enterococcus gilvus*, *Enterococcus casseliflavus*, *Segatella oris*, and *Akkermansia muciniphila*. Serum zonulin levels were higher in CRC patients (CRC = 70.1 ± 26.14, Control = 53.93 ± 17.33, *p* = 0.048). There is a significant relationship between gut microbiota and CRC. A multifactorial evaluation of this relationship could shed light on potential biomarker identification and the development of new treatment options for CRC.

## 1. Introduction

According to the WHO 2022 data, approximately 1.9 million new cases of colorectal cancer (CRC) and 882,000 related deaths were reported [1]. Colorectal cancer ranks third in incidence among the most common cancers, accounting for 10.6% in men, following lung and prostate cancer, and 9.5% in women, second to breast cancer [2]. Diet, environmental factors, and genetic predisposition play roles in CRC etiology [1,2]. Early diagnosis is crucial for curative treatment in CRC. Individuals identified symptomatically or through screening should undergo colonoscopy or flexible sigmoidoscopy with concurrent biopsy for definitive diagnosis [3]. CT colonography and the presence of Streptococcus bovis in blood culture are utilized [4]. The discovery of new microbial biomarkers through microbiota analysis will also contribute to screening efforts.

The microbiota can colonize nearly every surface of the human body, primarily in the gastrointestinal (GI) tract but also in the genitourinary system, respiratory tract, and skin, interacting with the external environment [5]. It is estimated that the colon alone harbors over 70% of all microbes in the human body [5]. Microbial distribution varies not only between individuals but also within individuals due to factors such as genetics, age, sex, pregnancy, diet, geography, and antibiotic use [6]. Diet is one of the most important factors influencing the gut microbiota. A Western-style diet, characterized by high sugar and fat intake and low consumption of fruit and vegetable fibers, reduces beneficial *Firmicutes* levels while increasing *Proteobacteria* [7]. In contrast, the Mediterranean diet, which consists of whole grains, nuts, vegetables, fruits, and selected animal products such as fish and poultry, has shown beneficial effects on the host. The addition of prebiotics to the diet also exerts positive effects on the microbiota. Prebiotics are indigestible dietary fibers that increase the abundance of *Bifidobacterium* and *Lactobacillus*, thereby enhancing mucosal barrier integrity and host immunity while reducing the interaction of opportunistic enteric pathogens with the mucosa [8]. Microbiota, especially, has been implicated in the development of various diseases including cardiovascular, respiratory, brain, kidney, and liver diseases, as well as inflammatory bowel disease and diabetes [7].

The intestinal microbiota operates in harmony with the host intestinal mucosa, providing crucial metabolic, immunological, and protective functions. Among its metabolic roles are the metabolism of carbohydrates, proteins, bile acids, vitamins, and plant polyphenols. Colon bacteria primarily utilize dietary carbohydrates as nutrients, resulting in the production of short-chain fatty acids (SCFAs) and gases through fermentation. The most abundant SCFAs detected in feces are acetate, propionate, and butyrate. Butyrate is the most important SCFA for human health, serving as a primary energy source for colonocytes and regulating gene expression by inducing apoptosis in colon cancer cells and inhibiting histone deacetylases, thus potentially possessing anti-cancer activity [9]. Propionate acts as an energy source for epithelial cells, contributes to gluconeogenesis when transferred to the liver, and induces satiety signals [10]. Acetate is the most abundant SCFA and serves as a necessary cofactor for the growth of other bacteria, being transported to peripheral tissues in the human body where it is involved in cholesterol metabolism and lipogenesis. It also contributes to intestinal homeostasis and inhibits CRC progression [11]. These SCFAs are produced by specific bacteria: butyrate is mainly produced by the *Lachnospiraceae* family and *Faecalibacterium prausnitzii* species, while propionate is produced by *Bacteroides* and some *Clostridium* species [10].

The intestinal microbiota also plays a significant role in the development of intestinal mucosal and systemic immunity. Lipopolysaccharides (LPS), found in the cell walls of Gram-negative bacteria and also known as endotoxins, can enter the systemic circulation and trigger innate immune responses by penetrating into the liver and adipose tissue. Due to its enhanced inflammatory response, LPS can alter intestinal barrier function [12]. Zonulin is a 47 kDa human protein that can reversibly modulate intercellular tight junctions, necessary for maintaining the physiological processes in the intestine. Inflammatory bowel disease, dietary factors, and the microbiota activate the release of zonulin via CXCR3 [13]. Increased zonulin release leads to heightened intestinal permeability. Two significant factors triggering zonulin release are gluten and microbial overgrowth [14].

Multifactorial studies encompassing microbiota, microbiota metabolites, and zonulin are severely limited in colorectal cancer patients. This study examined the intestinal microbiota profiles of CRC patients and healthy controls, along with fecal SCFA levels, serum zonulin and lipopolysaccharide-binding protein (LBP) levels. It evaluated multifactorially the intestinal dysbiosis and associated metabolite changes occurring in CRC patients. The findings are expected to contribute to the identification of potential biomarkers for CRC diagnosis and possible pro/prebiotic candidates for therapeutic use, thereby supporting the development of cost-effective strategies in healthcare. Moreover, the data obtained from this study may provide valuable input for future meta-analyses in this field.

## 2. Materials and Methods

Between March 2023 and December 2023, 16 newly diagnosed and untreated CRC patients were included in the study from the General Surgery Outpatient Clinic of Selçuk University Faculty of Medicine. As a control group, 16 healthy individuals with negative CRC screening results were also included. The exclusion criteria for participation in the study were being under 18 years of age, pregnancy, antibiotic use or a history of surgical intervention within the past three months, the presence of chronic disease, and active infection.

For the study, microbiota and SCFA analyses were planned from stool samples, while LBP and zonulin analyses were planned from serum samples. Stool samples (1 g each) obtained from participants were transferred into two separate sterile screw-cap tubes and labeled with barcodes. Blood samples from the same individuals were collected into serum separator tubes. After allowing the samples to clot at room temperature, they were centrifuged at 3000× *g* for 10 min, and the serum phase was separated. The obtained serum samples (≥1 mL) were transferred into Eppendorf tubes and barcoded. All samples were stored at −80 °C until analysis.

In our study, DNA isolation from stool samples for microbiota analysis was initially performed using the Quick-DNA™ Fecal/Soil Microbe Miniprep Kit (Zymo Research, Avenue, Irvine, CA, USA). During the isolation process, approximately 150 mg of stool sample was transferred into lysis tubes, homogenized with buffer, subjected to brief incubation at 90 °C, and following centrifugation, the resulting supernatant was processed through column-based purification steps to obtain DNA. From the isolated DNA, amplification targeting approximately 1500 bp region spanning the V1–V9 regions of the 16S rRNA gene was carried out using a primer pair. The amplification process was conducted using the MyGeneTM L Series Peltier Thermal Cycler (LongGene, Hangzhou, China) set to specific thermal cycles. Amplified products were prepared and sequenced using Oxford Nanopore Technologies’ ligation-based library preparation kits for third-generation long-read sequencing. Library preparation and sequencing were performed using the SQK-NBD114.96 kit and FLO-MIN114 spot-on flow cells on the Mk1C sequencing device (Oxford Nanopore Technologies, Oxford, UK).

After sequencing, the results obtained in fast5 format were converted to fastq format using Guppy software for bioinformatics analysis. Reads were cleaned using Trimmomatic and analyzed using the Python programming language. Each sequence was matched against a reference database using the Blast algorithm to obtain taxonomic data for sequences with over 60% coverage and 80% pairwise similarity, thereby generating Operational Taxonomic Units (OTUs).

The OTU file generated was then used for various analyses using different indices, including alpha diversity analysis, PCA (Principal Component Analysis), PCoA (Principal Coordinates Analysis), beta diversity analysis, phenotype analyses, and biomarker discovery using LEFse analysis.

Serum zonulin and LBP levels were measured using E-EL-H5560 and E-EL-M3090 ELISA kits (Elabscience, Houston, TX, USA) following the sandwich ELISA principle. Short-chain fatty acid analysis was conducted using a GC-MS/MS (Gas Chromatography-Mass Spectrometry/Tandem Mass Spectrometry) device equipped with a flame ionization detector and a DB-FFAP column (Agilent 8860 Series, Memphis, TN, USA). For this procedure, 1 g of stool sample was mixed with 2 mL of distilled water and vortexed for 10 min. The mixture was centrifuged at 10,000× *g* for 1 min at room temperature to obtain the supernatant. A 1 µL aliquot of the supernatant was injected into the injector chamber, where it rapidly evaporated and was carried into the column by the carrier gas hydrogen. Fatty acids were retained in the column according to their carbon chain length and subsequently reached the detector, where they were ionized. The resulting ions were converted into electrical signals proportional to their abundance, which were transferred to the OpenLAB CDS data processor to generate chromatograms. Peak areas and retention times were then calculated from the chromatograms, compared with standard samples, and the percentage of each fatty acid was determined.

### Statistical Analysis

Statistical analysis was performed using R version 4.2.1 software. Numerical variables were presented as mean ± standard deviation, while categorical variables were presented as frequency (n) and percentage (%). The average ages of colorectal cancer patients and healthy controls were compared using Student’s t-test, and gender distributions were compared using Yates continuity-corrected chi-square test. Additionally, differences in the detection rates of taxa at the phylum, class, family, genus, and species levels between CRC patients and healthy controls, as well as differences in levels of fatty acids, zonulin, and LBP, were assessed using the Mann–Whitney U test. A significance level of 5% was used for all analyses.

## 3. Results

16 patients diagnosed with colorectal cancer (8 female, 8 male) and 16 healthy controls (8 female, 8 male) were included in the study, ensuring age and gender matching.

Alpha diversity refers to the diversity of species present within a given sample. Alpha diversity in the groups was calculated using Shannon, Simpson, and Chao-1 indices. According to Shannon and Simpson indices, intestinal microbial alpha diversity was significantly lower in CRC patients (*p* = 0.045 and *p* = 0.017, respectively). However, there was no significant difference in alpha diversity between groups according to the Chao-1 index (*p* = 0.24).

**Figure 1:**
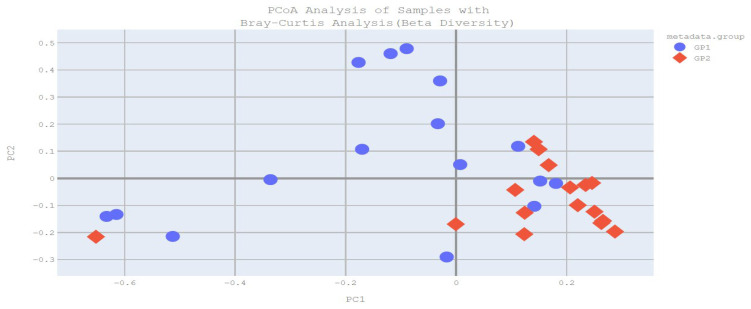
Bray–Curtis PCoA analysis plot (GP1: patient group, GP2: control group).

The primary objective of beta diversity analysis is to compare samples within themselves and across groups in order to observe structural differences between groups or individual samples. Beta diversity was calculated using Bray–Curtis PCoA analysis. According to Bray–Curtis PCoA analysis, a significant difference in intestinal microbial beta diversity was found between the groups (*p* = 0.004) (Figure 1).

*Bacillota* (*Firmicutes*) was the most commonly detected phylum in both groups at the phylum level. However, in CRC patients, this was followed by *Pseudomonadota* (*Proteobacteria*) and *Bacteroidota*, whereas in healthy controls, it was followed by *Bacteroidota* and *Pseudomonadota* (*Proteobacteria*). The detection rate of *Bacteroidota* was statistically significantly lower in CRC patients compared to healthy controls (*p* = 0.027). Although not reaching statistical significance, an increased detection rate of *Pseudomonadota* (*Proteobacteria*) was observed in CRC patients (*p* = 0.258). However, similar detection rates of *Bacillota* (*Firmicutes*), *Verrucomicrobiota*, and *Actinomycetota* were found between CRC patients and healthy controls (*p* = 0.985, *p* = 0.074, *p* = 0.067, respectively) (Table 1).

When the intestinal microbiota composition was examined at the class level in the study groups, the most common classes identified in both groups were *Clostridia*, *Bacilli*, *Bacteroidia*, *Gammaproteobacteria*, *Erysipelotrichia*, *Verrucomicrobiae*, and *Negativicutes*. In colorectal cancer patients, the detection rates of *Clostridia*, *Bacteroidia*, and *Negativicutes* were significantly lower compared to healthy controls (Respectively, *p* = 0.035, *p* = 0.027, *p* = 0.016).

In colorectal cancer patients, the detection rate of *Lachnospiraceae* at the family level was significantly lower compared to healthy controls (*p* < 0.001), while the detection rate of *Enterococcaceae* was statistically significantly higher (*p* = 0.043). Although not reaching statistical significance, it was observed that the detection rate of *Enterobacteriaceae* was increased in colorectal cancer patients compared to healthy controls (*p* = 0.196).

In the intestinal microbiota of study groups at the genus level, *Enterococcus* detection rate was found to be significantly higher in CRC patients compared to healthy controls (*p* = 0.043). Moreover, *Roseburia*, *Lachnoclostridium*, and *Blautia* detection rates were significantly lower in colorectal cancer patients compared to healthy controls (respectively, *p* = 0.002, *p* = 0.025, *p* < 0.001). Although not reaching statistical significance, it was observed that the detection rates of *Escherichia* and *Akkermansia* were increased in CRC patients compared to healthy controls (respectively, *p* = 0.138, *p* = 0.074) (Table 2).

When the intestinal microbiotas of the study groups were examined at the species level, detection rates of *Enterococcus faecium* and *Ruminococcus* bicirculans were found to be statistically significantly higher in CRC patients compared to healthy controls (*p* = 0.04 and *p* = 0.027, respectively). *Enterococcus gilvus*, *Enterococcus casseliflavus*, *Segatella oris*, and *Alistipes senegalensis* species were not detected in healthy controls but were detected at higher rates in CRC patients (*p* = 0.039, *p* = 0.019, *p* = 0.019, *p* = 0.039). Although not reaching statistical significance, increased detection rates of *Escherichia coli* and *Akkermansia muciniphila* species were observed in CRC patients compared to healthy controls (*p* = 0.138, *p* = 0.074) (Table 3).

**Figure 2:**
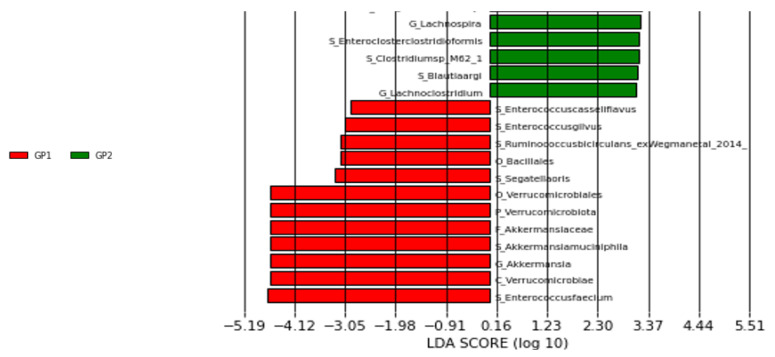
LEFse plot of taxa at genus and species levels (GP1: patient group, GP2: control group).

In our study, LEFse analysis revealed 6 different species with LDA scores > 2: Enterococcus faecium, Enterococcus gilvus, Enterococcus casseliflavus, Ruminococcus bicirculans, Segatella oris, and Akkermansia muciniphila (Figure 2).

When serum zonulin and LBP levels were examined in the study groups, zonulin levels were found to be statistically significantly higher in CRC patients compared to healthy controls, while LBP levels, although not reaching statistical significance, were higher in CRC patients (*p* = 0.048 and *p* = 0.171, respectively) (Table 4).

When fecal short-chain fatty acids were examined in the study groups, acetate, propionate, and butyrate were found to be decreased in CRC patients, although these changes did not reach statistical significance (*p* = 0.128, *p* = 0.086, *p* = 0.423, respectively) (Table 5).

## 4. Discussion

The term ‘oncomicrobiome’ was introduced by the International Cancer Microbiome Consortium to describe the characteristic microbiota profile that differs in oncological patients compared to healthy individuals and could potentially become a non-invasive tool for early diagnosis, with dysbiosis defined as a condition contributing to cancer [15].

In a study by Gao et al. it was found that alpha diversity, as assessed by Shannon and Simpson indices, was significantly decreased in CRC patients compared to healthy controls, while no significant difference was observed between the two groups according to the Chao-1 index [16]. In another study by Tengfei et al. it was reported that alpha diversity, based on Shannon and Chao-1 indices, was significantly reduced in CRC patients compared to healthy controls [17]. A comprehensive meta-analysis encompassing 14 studies conducted in the USA revealed significant differences in beta diversity between CRC patients and healthy controls in the intestinal microbiota [18]. Consistent with the literature, our study found decreased alpha diversity and distinct beta diversity between CRC patients and healthy controls. These findings confirm that microbial diversity is reduced due to intestinal dysbiosis in cancer.

In a study investigating the relationship between CRC and intestinal microbiota, the *Bacillota* (*Firmicutes*) phylum was found to be significantly lower in CRC patients compared to healthy controls, and the *Bacteroidota* phylum was found to be higher. Additionally, although it did not reach a statistically significant level, it was determined that *Actinobacteria* decreased and *Pseudomonadota* (*Proteobacteria*) increased in CRC patients [19]. In the study conducted by Wang et al., it was found that *Pseudomonadota* (*Proteobacteria*) increased significantly and *Bacteroidota* decreased in CRC patients compared to healthy controls, but the detection rates of *Bacillota* (*Firmicutes*) were found to be similar between the groups [20]. In our study, the most detected phylum in both groups was *Bacillota* (*Firmicutes*), followed by *Pseudomonadota* (*Proteobacteria*) in CRC patients and *Bacteroidota* in healthy controls. *Bacteroidota* was found to be significantly lower in CRC patients compared to healthy controls. Additionally, *Pseudomonadota* (*Proteobacteria*) were detected more in CRC patients than in healthy controls, but it was found that this did not reach a significant level. In colorectal cancer, the bacterial balance is disrupted. This disrupted balance creates a basis for the proliferation of opportunistic pathogens, and tumor formation is observed as a result of a series of immunological and genetic events.

When assessed at the class level, Bamola et al. found in their study that *Bacilli* and *Actinobacteria* were significantly lower in the intestinal microbiota of CRC patients compared to healthy controls, whereas *Bacteroidia* and *Gammaproteobacteria* were higher [19]. In a study conducted in China, it was observed that *Clostridia* levels were lower in CRC patients compared to healthy controls, while *Gammaproteobacteria* and *Negativicutes* levels were higher [17]. In our study at the class level, we similarly found that *Clostridia*, *Bacteroidia*, and *Negativicutes* were significantly lower in CRC patients compared to healthy controls. *Clostridia* are a typical group of anaerobic bacteria known for their capacity to produce butyrate. Butyrate plays a role as a mediator in various metabolic and immune functions, contributing to the maintenance of intestinal homeostasis. Decreased levels of healthy intestinal microbial elements such as *Clostridia* and *Negativicutes* may shift the balance in favor of pathogenic microorganisms, potentially leading to dysbiosis and tumor formation [21].

When assessed at the family level, Tengfei et al. found in their study that *Lachnospiraceae* were significantly decreased, while *Enterobacteriaceae* were increased in the intestinal microbiota of CRC patients compared to healthy controls [17]. Another study in China reported increased levels of *Enterococcaceae*, *Peptostreptococcaceae*, and *Enterobacteriaceae* in the intestines of CRC patients [20]. In our study, we similarly found increased levels of *Enterococcaceae* and decreased levels of *Lachnospiraceae* in the intestinal microbiota of CRC patients. The detection rates of *Peptostreptococcaceae* and *Enterobacteriaceae* were also higher in CRC patients compared to healthy controls, although this difference did not reach statistical significance. *Lachnospiraceae* are one of the main taxonomic groups in the human intestinal microbiota, known for breaking down complex polysaccharides into short-chain fatty acids (SCFAs), and are considered major producers of butyrate. Butyrate is known for its anti-inflammatory effects and inhibitory effects on the proliferation of colon cancer cells [22,23]. The family *Enterococcaceae* includes some of the most common Gram-positive cocci in the intestine. Certain species within this family are known to produce superoxide, hydrogen peroxide, and hydroxyl radicals in mammalian cells, which can lead to DNA damage and contribute to CRC development [24]. These findings suggest that CRC development may stem from a pro-inflammatory process and disruption of the balance between the host and microbiota. Hypotheses also exist suggesting that dysbiosis may lead to CRC formation through systemic rather than purely local effects, given the similarity in microbiota between tumor sites and adjacent tumor-free mucosa [25].

In the study by Wang et al. an increase in *Enterococcus*, *Peptostreptococcus*, *Porphyromonas*, and *Escherichia*/*Shigella* genera in CRC patients was notably observed. Additionally, *Roseburia* and *Alistipes* genera were found to be lower in CRC patients in the same study [20]. Zhao et al. in a comprehensive study aggregating data from various studies involving 353 patients, found significant decreases in *Faecalibacterium*, *Alistipes*, and *Blautia* genera, and increases in *Fusobacterium* and *Campylobacter* genera in CRC patients compared to healthy controls [21]. In our study, we found increased levels of *Enterococcus* and decreased levels of *Roseburia*, *Blautia*, and *Lachnoclostridium* genera in CRC patients compared to healthy controls. Although not reaching statistical significance, we also observed an increase in *Akkermansia* and a decrease in *Faecalibacterium* in CRC microbiota compared to healthy controls. Some species of *Enterococcus* have been shown in experiments to increase extracellular superoxide release, which can convert to hydrogen peroxide, leading to chromosomal instability and DNA damage that may trigger carcinogenesis [26]. *Blautia*, a group of anaerobic bacteria within the *Lachnospiraceae* family, produces bacteriocins that can inhibit the colonization of certain pathogenic bacteria in the intestine, influencing the composition of the gut microbiota. This genus also exhibits potential probiotic properties [27]. *Roseburia*, another member of the *Lachnospiraceae* family, is one of the major producers of butyrate in the gut. Butyrate plays protective roles against intestinal infections and cancer through its anti-inflammatory and metabolic modulation effects. Due to these characteristics, *Roseburia* is considered a candidate for probiotic use [28].

In the study by Tengfei et al. it was found that *Faecalibacterium prausnitzii* was decreased and *Escherichia coli* and *Prevotella copri* were increased in the intestinal microbiota of CRC patients [17]. Another study reported a significant increase in *Akkermansia muciniphila* in the gut microbiota of CRC patients [29]. A study conducted in Iran focusing on CRC tumor tissue revealed increased levels of *Enterococcus faecalis* and enterotoxigenic *Bacteroides fragilis*, and decreased levels of *F*. *prausnitzii* compared to healthy controls [24]. Various studies have highlighted the increase in *E. faecalis*, *E. coli*, enterotoxigenic *Bacteroides fragilis* (ETBF), and *Peptostreptococcus anaerobius* in CRC patients [30]. In our study, six different species were identified as statistically significant. *Enterococcus faecium* and *Ruminococcus bicirculans* (ex Wegman et al. 2014 [31]) were found to be higher in CRC patients compared to healthy controls. *Enterococcus gilvus*, *Enterococcus casseliflavus*, *Segetella oris*, and *Alistipes senegalensis* were not found in healthy controls but were relatively increased in CRC patients. Although not reaching statistical significance, we observed increased detection rates of *E. coli* and *A. muciniphila*, and decreased detection rates of *F. prausnitzii* in CRC patients. Our LEFse analysis identified six different species with an LDA score > 2, potentially serving as biomarker candidates. These included statistically significant species such as *E. faecium*, *E. gilvus*, *E. casseliflavus*, *S. oris*, *R. bicirculans*, *and A. muciniphila.*

Various species of *Enterococcus* play a significant role in gut homeostasis by stimulating the immune system. While some strains are beneficial, others have been associated with diseases including CRC. *E. faecalis*, for instance, primarily induces colon cancer susceptibility by producing reactive oxygen species and extracellular superoxide, leading to genomic instability and mutations in colonic DNA [32]. Conversely, certain strains of *E. faecium* are predicted to be used as probiotics for lowering cholesterol levels and treating diarrhea and irritable bowel syndrome [33]. In a study on mice with CRC, administration of *E. faecium* CRL-183 suspension reduced adenocarcinoma incidence by 40% and decreased average tumor size in CRC-afflicted mice [34]. Further research is needed to understand the relationship between CRC and *Enterococcus* species such as *E. gilvus* and *E. casseliflavus*, which we identified as potential biomarkers but have not been extensively discussed in the literature.

*Ruminococcus* bicirculans belongs to the *Bacillota* (*Firmicutes*) phylum and was recently identified as a new species following completion of genomic analysis [31]. *Ruminococcus* species are involved in resistant starch digestion, amino acid biosynthesis, and lipid metabolism [35]. The benefits and risks of *Ruminococcus* are debated. It has been suggested that *Ruminococcus* could potentially be used as a probiotic that might yield positive outcomes in the treatment of hepatocellular carcinoma patients receiving anti-PD-1 immunotherapy. On the other hand, increased levels of *Ruminococcus* have been found in certain cancers, suggesting it could serve as a biomarker for prognosis [36]. In studies evaluating CRC patients, an increase in *R. bicirculans* has been shown, especially with age [37]. In our study, we observed an increase in *R. bicirculans* in CRC patients compared to healthy controls, and our analysis identified it as a potential biomarker candidate.

*S. oris* (*Prevotella oris*) is a Gram-negative anaerobic bacterium primarily found in the gut, mouth, and lower respiratory tract microbiota [38]. Oral pathogens like *S. oris* can translocate from the mouth to the gut. This can lead to gut dysbiosis, increasing the production of innate and acquired immune response elements. The inflammatory response results in the production of reactive nitrogen and oxygen species, oxidative stress, DNA damage, dysregulated cell proliferation, and ultimately, the formation of CRC [25]. *A. senegalensis* belongs to the *Bacteroidota* phylum, and its genome sequence has recently been completed. *Alistipes* species are typically found in healthy human gut microbiota and can produce short-chain fatty acids (SCFAs) from amino acids. Due to their association with various diseases, they are considered opportunistic pathogens. Increased levels of *Alistipes* have been noted in conditions such as cardiovascular diseases, colitis, depression, and CRC, particularly in right-sided colon tumors [39]. *Alistipes* is presumed to induce IL6-STAT3 signaling, triggering tumorigenesis [40]. In our study, we observed an increase in *S. oris* and *A. senegalensis* species in CRC patients compared to healthy controls. Our analysis suggests that *S. oris* could be a potential biomarker candidate, but further research is needed to confirm this.

*Akkermansia muciniphila* is a Gram-negative bacterium belonging to the *Verrucomicrobia* phylum, specialized in degrading mucin in the gut. An overabundance of mucin-degrading bacteria can weaken the mucosal barrier integrity and increase inflammation, which initiates the process of CRC formation [41]. In studies conducted on mice, oral administration of *A. muciniphila* led to shortened colons, greater body weight loss, and increased tumor burden compared to healthy controls [42]. However, conflicting results have emerged regarding the effects of *A. muciniphila* on CRC. Administering the DSM 22959 strain of *A. muciniphila* in colitic mice reduced disease activity index, increased mucus thickness, and enhanced anti-inflammatory activity, thereby accelerating recovery from colitis [43]. This variability in outcomes could be attributed to different strains of bacteria used for probiotic purposes having varying effects on microbiota in different body regions. In our study, although not reaching statistical significance, we observed an increased incidence of *A. muciniphila* in CRC patients. Our analysis suggests that *A. muciniphila* could be a potential biomarker candidate for CRC. Moreover, various studies in human and mouse models have shown that reducing food intake can significantly increase *A. muciniphila* or related bacteria. Given that decreased food intake is known in colorectal cancer patients, analyzing diet information is crucial for a meaningful interpretation of increased *A. muciniphila* levels [44]. Overall, these findings suggest that rather than causing CRC, increased levels of *A. muciniphila* in advanced CRC cases may be a response to restore the disrupted intestinal barrier.

*Escherichia coli* are Gram-negative facultative anaerobic bacteria belonging to the phylum *Pseudomonadota* (*Proteobacteria*), and they are predominantly found in the normal intestinal flora. They contribute to maintaining the stability of luminal microbial flora and preserving normal intestinal homeostasis [45]. Studies have indicated an increase in pathogenic *E. coli* strains belonging to the B2 phylogroup, particularly those producing colibactin, in CRC patients. Specifically, the pks+ *E. coli* (11G5) strain has been shown to significantly induce tumor formation by increasing the size and number of tumors in CRC [46]. Some studies have found an increased prevalence of pks+ *E. coli* in normal tissues and early adenomas, while a recent French cohort study reported enrichment of pks genes only in stage 4 tumors [47]. In our study, although not reaching statistical significance, we observed an increase in *E. coli* levels in CRC patients compared to healthy controls. Understanding the role of colibactin produced by *E. coli* in tumor formation and cancer progression requires further extensive research.

Tight junctions between enterocytes, which are composed of protein compounds, play a crucial role in intestinal barrier permeability. Dysbiosis occurring for any reason can lead to increased intestinal barrier permeability through inflammation. The discovery of zonulin, the human equivalent of the Zot toxin produced by *V. cholerae*, has shed light on the connection between disrupted intestinal barrier permeability and disease pathogenesis. In a study, opportunistic pathogens were found to increase in the gut microbiota of CRC patients with existing obesity, and it was shown that their serum zonulin levels were elevated compared to healthy controls [48]. In another study investigating the connection between colorectal cancer and zonulin levels, it was observed that zonulin levels increase as the disease progresses in CRC patients [49]. Administering probiotics to colorectal cancer patients has been shown to reduce serum zonulin levels. Particularly, postoperative serum zonulin levels have been proposed as an early biological marker for sepsis [50]. Similarly to the literature, our study found significantly elevated serum zonulin levels in CRC patients compared to healthy controls. These results indicate an increase in intestinal permeability correlating with zonulin levels, possibly due to intestinal microbial dysbiosis, leading to increased permeability and, concurrently, the initiation of CRC formation through the influence of various molecules. Serum zonulin levels, affected by various diseases, are considered for early diagnosis of sepsis in CRC, as pathogens may enter systemic circulation due to increased intestinal permeability. However, more comprehensive studies are needed for this purpose.

Lipopolysaccharides (LPS) are components of the cell wall of Gram-negative bacteria, while Lipopolysaccharide Binding Protein (LBP) is an acute-phase protein produced by hepatocytes in response to bacteria and LPS. LPS alters intestinal barrier function by increasing inflammatory responses. Various studies have found that decreased apoptosis and increased proliferation in metastatic tumor cells are associated with LPS and LBP as indicators of LPS exposure. In a study examining LPS levels in patients with colorectal adenomas, higher levels of LPS were found in adenoma patients compared to healthy controls [51]. It has been found that blood LBP levels exhibit a negative correlation with the *Clostridiales* order and a positive correlation with the *Bacteroidales* order. *Bacteria* in the *Clostridiales* order produce butyrate, which strengthens intestinal barrier function and suppresses LPS flow. *Bacteria* in the *Bacteroidales* order, however, generally damage the intestinal barrier due to metabolites they produce, leading to intestinal dysbiosis and shifting the balance in favor of Gram-negative opportunistic pathogens, thereby increasing LPS production [52]. Although not reaching statistical significance in our study, an increase in serum LBP levels was observed in CRC patients compared to healthy controls. Due to intestinal dysbiosis in colorectal cancer patients, there is a decrease in the *Bacillota* (*Firmicutes*) phylum and an increase in the *Pseudomonadota* (*Proteobacteria*) phylum, shifting the balance in favor of opportunistic Gram-negative bacterial groups. In our study, the probable reason for the increase in serum LBP levels is the increased opportunistic Gram-negative bacteria in the microbiota of CRC patients.

Short-chain fatty acids (SCFAs) are metabolites produced by the anaerobic gut microbiota through the fermentation of fibers, readily absorbed to provide energy to colon cells. The three main SCFAs in the intestines are butyrate, propionate, and acetate [53]. Butyrate is primarily produced by *Bacillota* (*Firmicutes*) and helps prevent tumor formation by reducing inflammation. Butyrate increases mucus secretion, thereby protecting the intestinal epithelial barrier, and acts as a histone deacetylase inhibitor (HDAC), inhibiting the Wnt/β-catenin signal and regulating apoptosis in CRC cells to prevent their proliferation. Propionate is produced by certain bacteria within the *Bacteroidota* phylum. Despite being less potent than butyrate, propionate serves as an HDAC inhibitor that inhibits malignant transformation and induces apoptosis in pre-cancerous colon cells. Studies have shown a decrease in propionate abundance in CRC patients [53]. In vitro experiments have demonstrated that adding propionate to treatment regimens suppresses CRC progression [54]. Acetate is produced by *Propionibacterium* and *Bacteroides* groups, contributing to intestinal homeostasis and inhibiting CRC progression [53]. Isovalerate, formed from the fermentation of branched-chain amino acids like leucine, is considered harmful to the intestines. Research has shown that isovalerate causes relaxation in colonic smooth muscle via the cAMP/PKA pathway [55]. Emerging evidence suggests that isobutyrate may enhance metastasis in CRC via RACK1 activation [56].

In a study conducted in China, significant decreases in butyrate, acetate, and propionate levels were shown in patients with colorectal adenomas [57]. Various studies evaluating CRC patients have observed decreased levels of SCFAs (butyrate, acetate, propionate) [58]. In a different study in Italy, decreased acetate and propionate levels were observed in CRC patients, while valerate, isovalerate, and isobutyrate levels were found to be increased [59]. A study in the USA found decreased butyrate levels but increased valerate, isovalerate, and isobutyrate levels in CRC patients’ intestines [29]. Although in our study butyrate, acetate, and propionate did not reach statistically significant levels, they were relatively decreased in CRC patients. Levels of isobutyrate, isovalerate, valerate, isocaproic acid, hexanoic acid, and n-heptanoic acid were similar among groups. These findings, along with the taxonomic distribution of intestinal microbiota, were consistent with the results. Levels of *Roseburia* and *Faecalibacterium*, which are major producers of butyrate, were found to be decreased in CRC patients in our study, explaining the decrease in butyrate and highlighting its protective role in CRC. *Bacteroidota*, which produces propionate and acetate, also showed a significant decrease in the patient group in our study. Similarly, the decrease in these bacteria, which produce propionate and acetate, correlates with decreased SCFA levels and underscores their protective roles in CRC. Based on the data obtained in our study, it is believed that administering butyrate, acetate, and propionate to patients, especially butyrate, may reduce progression in CRC, as suggested in the literature.

Our study demonstrated that CRC patients exhibit reduced gut microbial diversity and a markedly different microbial composition compared with healthy controls. The relative abundance of Bacteroidota was significantly decreased in CRC patients, while *E. faecium*, *R. bicirculans*, *E. gilvus*, *E. casseliflavus*, *S. oris*, and *A. muciniphila* emerged as potential biomarker candidates. In addition, serum zonulin levels were significantly elevated, and although not statistically significant, SCFA levels showed a decreasing trend in CRC patients.

The association between the gut microbiota and CRC is strong, and rather than a unilateral perspective, a multifactorial approach that integrates intestinal permeability, microbial dysbiosis, and biochemical markers provides a more comprehensive understanding. Our findings indicate a shift in microbial balance toward opportunistic pathogens, supported by biochemical evidence. Given the high mortality and morbidity rates of CRC and its substantial economic burden on healthcare systems, the identification of non-invasive biomarkers and the development of potential prebiotic/probiotic candidates, in addition to existing invasive diagnostic tools, may offer cost-effective strategies. In this context, our study provides valuable insights for the identification of novel biomarkers and the development of innovative therapeutic approaches in CRC.

This study has several limitations. First, diet, one of the most important factors influencing the gut microbiota, was not included in the analysis. In addition, the gut microbiota is known to be shaped not only by individual variation but also by biogeographic distribution. Therefore, some discrepancies between our findings and those of previous studies may be attributable to geographic differences and the omission of dietary factors. The relatively small sample size further reduces the statistical power of the study and limits the generalizability of the results. To better elucidate cause–effect relationships, longitudinal monitoring of microbiota changes throughout the course of CRC and its treatment is warranted. Moreover, a clearer understanding of the association between gut microbiota and CRC will require large-scale, multifactorial studies.

## Figures and Tables

**Table 1:** Examination of the intestinal microbiota composition of the study groups at the phylum level.

Phylum (Relative Abundance %)	Study Groups		*p* Value
	Control (*n =* 16)	CRC (*n =* 16)	
*Bacillota* (*Firmicutes*)	71.36 ± 14.87	67.49 ± 26.24	0.985
*Bacteroidota*	19.74 ± 13.65	9.56 ± 12.09	**0.027**
*Pseudomonadota* (*Proteobacteria*)	7.11 ± 11.56	15.05 ± 24.16	0.258
*Verrucomicrobiota*	0.05 ± 0.15	7.19 ± 13.5	0.074
*Actinomycetota*	1.72 ± 2.48	0.68 ± 0.95	0.067

**Table 2:** Investigation of the gut microbiota composition of the study groups at the genus level.

Genus (Relative Abundance %)	Study Groups		*p* Value
	Control (*n =* 16)	CRC (*n =* 16)	
*Enterococcus*	4.11 ± 14.77	17.86 ± 34.23	**0.043**
*Salmonella*	0.09 ± 0.16	0.17 ± 0.25	0.280
*Clostridium*	2.52 ± 4.40	2.09 ± 3.56	0.571
*Roseburia*	1.88 ± 1.27	0.64 ± 1.22	**0.002**
*Prevotella*	0.12 ± 0.19	0.86 ± 2.20	0.911
*Escherichia*	5.21 ± 9.75	13.03 ± 21.18	0.138
*Segatella*	11.99 ± 12.22	4.08 ± 9.43	0.131
*Ruminococcus*	1.52 ± 1.14	1.33 ± 2.75	0.061
*Lachnoclostridium*	0.34 ± 0.19	0.16 ± 0.20	**0.025**
*Alistipes*	0.25 ± 0.30	0.44 ± 0.93	0.619
*Akkermansia*	0.05 ± 0.15	7.19 ± 13.50	0.074
*Faecalibacterium*	6.88 ± 8.32	2.94 ± 3.42	0.105
*Blautia*	15.84 ± 9.88	4.78 ± 5.46	**<0.001**

**Table 3:** Examination of intestinal microbiota composition at the species level in study groups.

Species (Relative Abundance %)	Study Groups		*p* Value
	Control (*n =* 16)	CRC (*n =* 16)	
*Enterococcus faecium*	3.95 ± 14.63	15.40 ± 31.48	**0.040**
*Enterococcus gilvus*	0.00 ± 0.00	0.01 ± 0.26	**0.039**
*Enterococcus casseliflavus*	0.00 ± 0.00	0.06 ± 0.11	**0.019**
*Ruminococcus bicirculans*	0.32 ± 0.37	0.35 ± 0.91	**0.027**
*Segatella oris*	0.00 ± 0.00	0.11 ± 0.20	**0.019**
*Alistipes senegalensis*	0.00 ± 0.00	0.03 ± 0.05	**0.039**
*Bacteroides fragilis*	0.21 ± 0.51	0.29 ± 1.14	0.200
*Escherichia coli*	5.08 ± 9.46	12.28 ± 19.79	0.138
*Faecalibacterium prausnitzii*	6.12 ± 7.92	2.66 ± 3.19	0.113
*Akkermansia muciniphila*	0.05 ± 0.15	7.19 ± 13.50	0.074

**Table 4:** Examination of serum zonulin and LBP levels in study groups.

Marker	Study Groups		*p* Value
	Control (*n =* 16)	CRC (*n =* 16)	
Zonulin (ng/mL)	53.93 ± 17.33	70.10 ± 26.14	**0.048**
LBP (ng/mL)	78.35 ± 33.11	94.89 ± 33.54	0.171

**Table 5:** Examination of fecal short-chain fatty acid levels in study groups.

Fatty Acid (µmol/mL)	Study Groups		*p* Value
	Control (*n =* 16)	CRC (*n =* 16)	
Acetate	8.66 (7.14–13.77)	7.30 (5.28–8.72)	0.128
Propionate	2.22 (1.46–4.03)	1.26 (0.89–2.50)	0.086
Butyrate	1.01 (0.67–2.27)	0.99 (0.50–1.85)	0.423
Isobutyric acid	0.30 (0.30–0.40)	0.23 (0.17–0.38)	0.509
Isovaleric acid	0.36 (0.26–0.51)	0.25 (0.18–0.47)	0.250
Valeric acid	0.14 (0.10–0.62)	0.12 (0.10–0.38)	0.610
Iso-caproic acid	0.10 (0.10–0.10)	0.10 (0.10–0.10)	0.072
Hexanoic acid	0.10 (0.10–0.18)	0.10 (0.10–0.10)	0.436
n-Heptanoic acid	0.10 (0.10–0.10)	0.10 (0.10–0.10)	0.576

## Data Availability

The original contributions presented in this study are included in the article. Further inquiries can be directed to the corresponding author.
